# Radiographic and perioperative outcomes following anterior thoracic vertebral body tethering and posterior lumbar spine tethering: a pilot series

**DOI:** 10.1007/s43390-023-00717-7

**Published:** 2023-06-25

**Authors:** Jeremy W. Siu, Hao-Hua Wu, Satvir Saggi, Sachin Allahabadi, Toshali Katyal, Mohammad Diab

**Affiliations:** 1 San Francisco School of Medicine, University of California, San Francisco, CA USA; 2 Department of Orthopaedic Surgery, University of California, San Francisco, CA USA

**Keywords:** Adolescent idiopathic scoliosis, Vertebral body tethering, Posterior lumbar spine tethering, Cable breakage, Revision operation

## Abstract

**Background and context:**

In patients with adolescent idiopathic scoliosis (AIS) of main thoracic and lumbar spine regions, combined anterior thoracic vertebral body tethering and posterior lumbar spine tethering (ATVBT/PLST) is a novel non-fusion treatment option for growth modulation and conservation of motion.

**Methods:**

Fourteen patients with AIS who underwent ATVBT/PLST with at least 2-year follow-up were included. Primary outcomes included quality of life as assessed by SRS-22 instruments, radiographic analysis, and revision operations. We secondarily reported perioperative metrics and post-operative opiate morphine equivalents (OME). Clinical success was defined as patients who achieved skeletal maturity with ≤ 30° curve magnitude of both their main thoracic and thoracolumbar/lumbar curves and who did not undergo posterior spine instrumentation and fusion (PSIF).

**Results:**

Patients had a mean age of 11.6 years (range 10–14 years), majority were girls (92%), and mean follow-up was 3.0 years (range 2–4.8 years). All patients were skeletally immature with a Risser ≤ 2. Included curves were Lenke 1C, 3C, or 6C. Mean preoperative curve magnitudes were 53° ± 8° (range 45°–65°) main thoracic and 49° ± 9° (range 40°–62°) thoracolumbar/lumbar curves. At most recent follow-up, patients had a mean main thoracic curve of 29° ± 8° (range 15°–40°) and a mean thoracolumbar/lumbar curve of 20° ± 15° (range 4°–35°). 50% required a revision operation. Cable breakage occurred in 43%, which did not always require revision. One patient progressed to thoracic fusion, but no patient underwent lumbar fusion. Patients had a mean SRS-22 outcome score of 4.2 ± 0.4.

**Conclusions:**

ATVBT/PLST is a potential alternative to spine fusion for select immature patients with AIS at a minimum 2-year follow-up. ATVBT/PLST potentially offers motion conservation at the cost of a higher revision rate. Further study and reporting of results are necessary to refine indications and techniques, which in turn will improve outcomes of this procedure.

**Level of evidence:**

Level IV—Case series without comparative group.

## Introduction

The standard surgical treatment for AIS is posterior spine instrumentation and fusion (PSIF). Recently, spine tethering has become a treatment option for a select cohort of skeletally immature patients, to conserve spine motion [1]. Although PSIF is effective, it eliminates motion and growth [2–6]. Spine tethering is a non-fusion approach that conserves motion and may guide growth to correct deformity in the child [1, 2, 7–10]. AIS patients undergoing thoracic PSIF with an additional thoracolumbar/lumbar structural curve or skeletally immature patients who are at risk for progression or junctional phenomena may require extension into the lumbar spine [11–14]. Tethering candidates are at risk for lumbar decompensation or developing a future lumbar curve due to significant growth remaining [11–13, 15–18]. Posterior lumbar spine tethering (PLST) conserves mobility and has the potential to allow for growth modulation compared with PSIF [2, 19–22]. PLST conserves spine motion and has the potential to allow for growth modulation compared with PSIF. It has the potential to improve the sagittal plane by restoring lordosis through compression by tensioning the cable between screws, which effect would be amplified by concave growth. By comparison, in vertebral body tethering (VBT), convex compression with concave growth is advantageous in the thoracic spine by being kyphosing but potentially disadvantageous in the lumbar spine. PLST also is lower morbidity compared with fusion [23–25].

We report clinical and radiographic outcomes of a novel treatment approach for skeletally immature patients with thoracic and lumbar scoliosis, treated by combined anterior thoracic vertebral body tethering and posterior lumbar spine tethering (ATVBT/PLST).

## Materials and methods

**Fig. 1:**
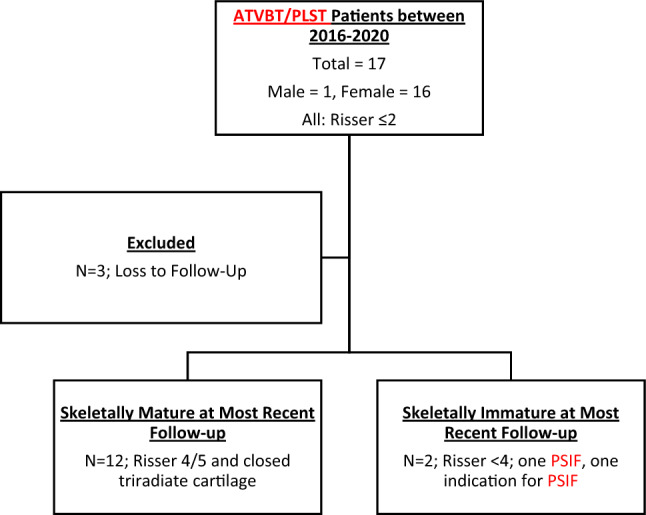
Flowchart of study population. *ATVBT/PLST* anterior thoracic vertebral body tethering and posterior lumbar spine tethering, *PSIF* posterior spine instrumentation and fusion

Following approval from the institutional review board (IRB), we conducted a single-center and single-surgeon retrospective cohort study of skeletally immature patients with idiopathic scoliosis who underwent ATVBT/PLST. Inclusion criteria were skeletally immature patients (Risser ≤ 2), main thoracic curve and lumbar curve ≥ 40°, combined ATVBT/PLST and a minimum of 2-year follow-up. Patients with Lenke 1C lumbar curves ≥ 40° standing but ≤ 25° bending were instrumented with posterior lumbar tethering if there was concern for distal adding on phenomenon [11–14]. Seventeen patients were available, of which 14 met inclusion criteria (Fig. 1). Patients with prior spine surgery, neuromuscular or syndromic scoliosis, history of infection, tumor or trauma were excluded.

Patient demographics included age at index operation, sex, menarchal status (for female patients), height, weight, BMI, curve magnitudes, Risser staging, triradiate cartilage, and Lenke classification.

Perioperative data included levels instrumented with anterior and posterior tether, operative complications, American Society of Anesthesiology (ASA) classification, case duration, estimated blood loss (EBL), removal of Foley catheter, days to solid food, length of stay. Perioperative opiate morphine equivalent (OME) use was recorded and converted to mg/kg to account for differences in weight.

Post-operative assessment included revision procedure and 30-day readmission. Functional outcomes were assessed with the Scoliosis Research Society 22-item Questionnaire (SRS-22).

Radiographic data were recorded preoperatively, as well as at 1st-, 2-year, and most recent post-operative visits. We measured curve magnitude by the standard method of Cobb at all time points, including in the event of cable breakage. Other radiographic measurements were thoracic kyphosis (sagittal view, T5–T12), lumbar lordosis (sagittal view, L1–S1), coronal imbalance (C7–CSVL), and shoulder height differences. For ATVBT/PLST patients who progressed to fusion, measurement data were not included in comparative analysis. To evaluate for cable breakage, we measured curve magnitude and inter-screw distance. We assumed cable breakage if there was progression as > 5° for an instrumented curve, consistent with spine deformity literature, and additional separation of screw heads > 2 mm compared with immediate surgical result.

Primary outcomes included quality of life as assessed by SRS-22 instruments, radiographic analysis and revision operations. Secondary outcomes included perioperative metrics and total post-operative OME use. Consistent with previous literature, clinical success was defined as patients who achieved skeletal maturity and ≤ 30° magnitude of both the main thoracic and lumbar curves at most recent post-operative visit [4]. Subsequent PSIF was considered a clinical failure.

Statistical analysis was performed in R (The R Foundation) v4.0.2 and RStudio v.1.3.1093. We expressed continuous data as mean ± standard deviation. Dependent t-tests were utilized to compare radiographic data over time. Dichotomous variables and categorical variables are expressed as values with percentages. The threshold of statistical significance was set at *α* = 0.05.

### Surgical technique

**Fig. 2:**
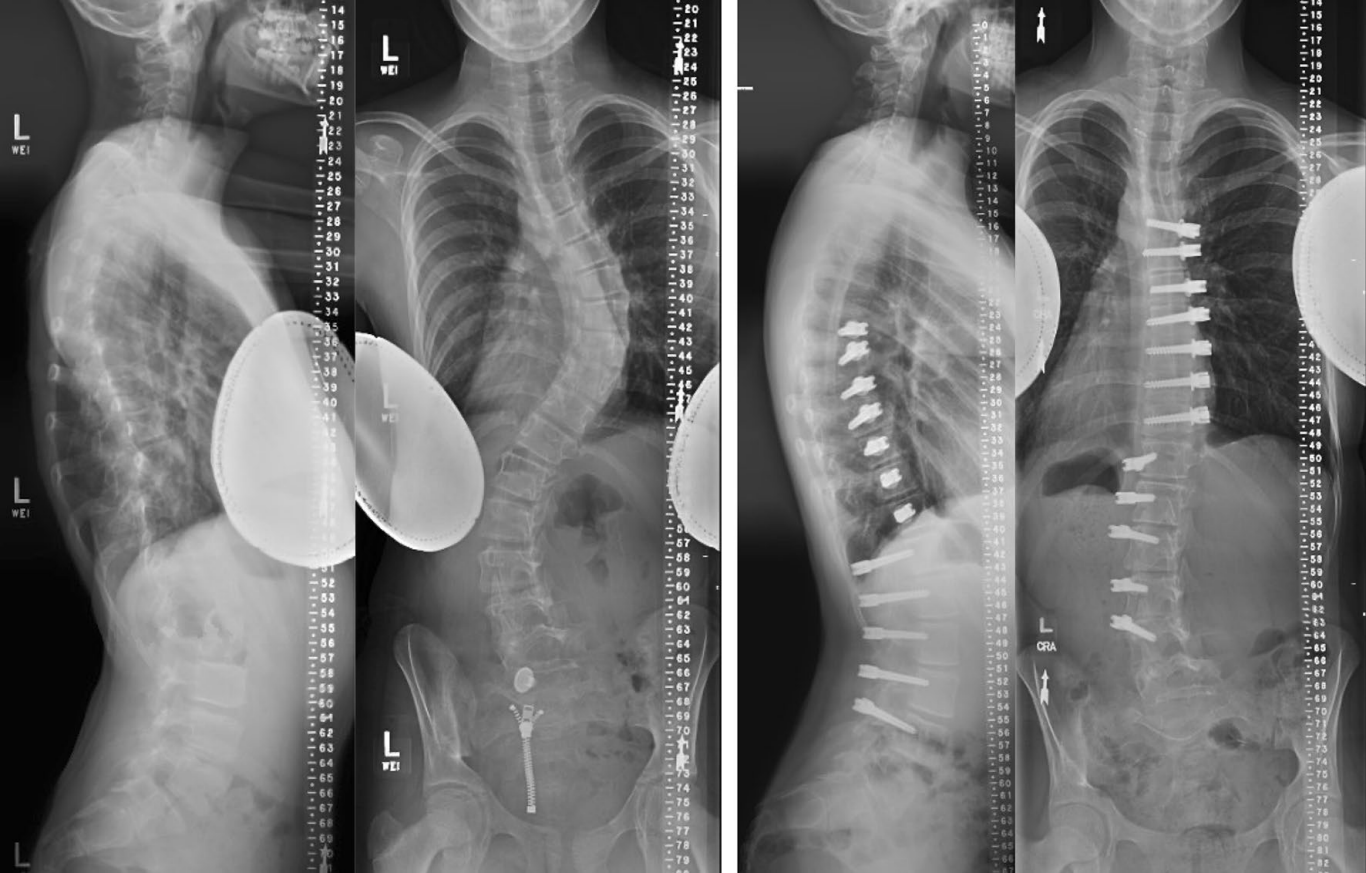
Patient with Lenke 3C classification with pre-operative 61o main thoracic curve and 47o lumbar curve (left images). Patient had an anterior tether from T5 to T11 and posterior tether from T12 to L4. At final post-op (right images), both curves have remained stable, and the patient is considered a clinical success

The senior author performed all ATVBT/PLST procedures at a single institution (Fig. 2). Selection of curves was based on radiographic magnitude and patient immaturity. Standard Stryker^®^ multaxial pedicle screws and rods were used for fusion, and Zimmer Biomet tethering system were used for both anterior and posterior tethering. Upper (UIV) and lower (LIV) instrumented vertebrae were selected as stable in coronal and sagittal planes based upon criteria established for spine fusion [26].

For ATVBT, an open muscle sparing approach was used on the side of curve convexity. This approach has been described in a previous study [27]. For posterior lumbar tethering, a Wiltse approach was used toward the curve convexity [28, 29]. Facet joints were spared in the dissection. Pedicle screws were started at the base of transverse process for a far lateral to medial trajectory, to avoid violating the facet joint and to keep screw heads away from this motion segment. The cable was attached by set screws, tightening sequentially to partially correct the deformity, to allow for future growth to continue correction and to guard against overcorrection.

Post-operative management included admission for pain control and mobilization, as well as a brace for 3 months to maximize the effect of the hydroxyapatite coating of the tethering screws.

## Results

### Patient demographics

Fourteen patients with AIS who underwent ATVBT/PLST were included in this study. Patient characteristics are summarized in Table 1. Mean age was 11.6 ± 1.7 years (range 10–14 years), 93% were girls, and mean follow-up time was 3.0 years (range 2–4.8 years). Mean preoperative height was 154.6 cm and 162.3 cm at most recent follow-up. Preoperative Risser staging distribution for 0/1/2/3/4/5 was 72%/ 14%/ 14%/ 0% / 0%/ 0% and post-operatively was 0%/ 7%/ 0%/ 7%/ 50%/ 36%. Preoperatively, 64% patients had an open triradiate cartilage and 7% post-operatively. Lenke classifications among patients were 1C (28%)/3C (43%)/6C (29%).

**Table 1:** Demographics of anterior thoracic vertebral body tethering and posterior lumbar spine tethering patients

	ATVBT/PLST (*N* = 14)
Age at index surgery	11.6 ± 1.7
Sex Female (%): Male (%)	13 (93%): 1 (7%)
Females premenarchal (*N* = 13)	11 (85%)
Follow-up (years)	3.0 ± 1.1 (2–4.8)
Preoperative data	
Height (cm)	154.6 ± 9.7
Upper Thoracic Cobb Angle	12 ± 6 (6 to 21)
Main Thoracic Cobb Angle	52 ± 8 (45–65)
Thoracolumbar/Lumbar Cobb Angle	49 ± 9 (40–62)
Risser	
0	10 (72%)
1	2 (14%)
2	2 (14%)
Triradiate Cartilage Open (%): Closed (%)	9 (64%): 5 (36%)
Post-operative Data	
Height (cm)	162.3 ± 9.1
Risser	
1	1 (7%)
3	1 (7%)
4	7 (50%)
5	5 (36%)
Triradiate Cartilage Open (%): Closed (%)	1 (7%): 13 (93%)
Lenke Classification	1C: 4 (28%) 3C: 6 (43%) 6C: 4 (29%)

Means with standard deviations are provided

Significant values are defined as *p* < 0.05

### Comparison of spine curvatures, coronal imbalance, and shoulder height differences in the ATVBT/PLST group

Cobb angles (main thoracic and thoracolumbar/lumbar) were recorded preoperatively and compared at 1st-, 2-year and most recent post-operative visits (Table 2). Main thoracic curve magnitude decreased significantly from mean 52° ± 8° (range 45°–65°) preoperatively to 25 ± 5° (range 13°–33°) at 1st post-operative visit, representing a 51% correction, 25° ± 14° (range 8°–30°) at 2-year post-operative visit (*p* < 0.001) and 29° ± 8° (range 15°–40°) at most recent post-operative visit (*p* < 0.001), representing a 43% correction. Similarly, the thoracolumbar/lumbar curve decreased from an average 49° ± 9° (range 40°–62°) preoperatively to 20° ± 10° (range 4°–30°) at 1st post-operative visit, representing a 59% correction, 15° ± 9° (range 5°–25°) at 2-year post-operative visit (*p* < 0.001) and 20° ± 15° (range 4°–35°) at most recent post-operative visit (*p* < 0.001), representing a 60% correction. There were no statistically significant changes to kyphotic angle. Lordotic curvature remained stable across preoperative, as well as 1st-, 2-year and most recent post-operative visits (*p* > 0.05).

**Table 2:** Comparison of major Cobb angles, coronal imbalance, and shoulder height difference in the anterior thoracic vertebral body tethering and posterior lumbar spine tethering groups at pre-op, first post-op, 2-year post-op and most recent post-op visits

	Preop		1st Erect post-op		*p*		2-year post-op		*p* value		Most recent post-op		*p* value
Upper Thoracic (degree)	12 ± 6 (6–21)		8 ± 3 (3–15)		**0.001**		8 ± 5 (4–23)		**0.01**		6 ± 2 (4–9)		**0.01**
Main Thoracic (degree)	52 ± 8 (45–65)		25 ± 5 (13–33)		** < 0.001**		25 ± 14 (8–30)		** < 0.001**		29 ± 8 (15–40)		** < 0.001**
Thoracolumbar/Lumbar (degree)	49 ± 9 (40–62)		20 ± 10 (4–30)		** < 0.001**		15 ± 9 (5–25)		** < 0.001**		20 ± 11 (4–35)		** < 0.001**
Kyphosis (T5–12) (degree)	21 ± 10 (4–42)		19 ± 11 (4–38)		0.506		21 ± 15 (3–40)		0.855		30 ± 12 (14–47)		0.11
Lordosis (L1–S1) (degree)	43 ± 9 (25–66)		40 ± 9 (26–49)		0.176		44 ± 8 (28–54)		0.841		45 ± 9 (30–63)		0.247
Coronal Imbalance (cm)	0.2 ± 2.5 (0–3.42)		0.4 ± 2.5 (0–4.4)		0.772		0.5 ± 1.5 (0–2.17		0.251		0.03 ± 1.6 (0–2.17)		0.871
Shoulder Height (cm)	0.7 ± 0.6 (0–2.1)		1 ± 1 (0–2.9)		0.12		1.1 ± 0.7 (0.6–2.4)		**0.03**		0.9 ± 0.7 (0.2–2.1)		0.487

Means with standard deviations are provided

Range is included in parentheses. One patient was excluded from 2-year and most recent follow-up measurements due to progression to fusion at 12 months. A second patient was excluded from most recent follow-up measurements due to progression to fusion at 40 months

Bold values are the significant variables, *p* < 0.05

We compared coronal imbalance and shoulder height differences between preoperative and all post-operative visits. Coronal imbalance did not show significant differences (*p* > 0.05). Shoulder height significantly increased from average preoperative height of 0.7 ± 0.6 cm to 1.1 ± 0.7 cm at 2 years (*p* = 0.03) but was not found to be significant at most recent follow-up.

### Perioperative metrics following ATVBT/PLST

Perioperative metrics were recorded following ATVBT/PLST (Table 3). Mean number of levels tethered was 11.8 with an average of 7.2 for anterior tether and 5.1 for posterior tether. For ATVBT, T5 was the upper instrumented vertebrae (UIV) in 8/14 (57%) patients and the lowest instrumented vertebrae (LIV) was T12 in 8/14 (57%) patients. In PLST, the UIV was T12 in 8/14 (57%) and L4 was the LIV in 8/12 (67%) of patients. There were no intraoperative complications and no 30-day readmissions.

**Table 3:** Perioperative metrics following anterior thoracic vertebral body tethering and posterior lumbar spine tethering

	TAVBT/PLST (*N* = 14)
Tethered levels	
Total	11.9 ± 0.5
Anterior thoracic	7.6 ± 0.8
Posterior lumbar	5.1 ± 0.7
Upper instrumented vertebrae (UIV)	
Anterior: T4 UIV	4 (28%)
Anterior: T5 UIV	8 (57%)
Anterior: T6 UIV	2 (14%)
Posterior: T10 UIV	1 (7%)
Posterior: T11 UIV	4 (28%)
Posterior: T12 UIV	8 (57%)
Posterior: L1 UIV	1 (7%)
Lowest instrumented vertebrae (LIV)	
Anterior: T10 LIV	1 (7%)
Anterior: T11 LIV	5 (35%)
Anterior: T12 LIV	8 (57%)
Posterior: L3 LIV	4 (28%)
Posterior: L4 LIV	10 (72%)
Intraoperative Complications	0%
ASA Classification	
1	8 (57%)
2	6 (43%)
Overall Length of Stay (days)	
Overall Length of Stay (days)	4.1 ± 0.6
Case length (min)	418.9 ± 111.3
Estimated Blood Loss (mL)	173.3 ± 81.2
Foley duration (h)	57 ± 17
Days to solid food	1.1 ± 0.8
30-day readmission	0%
Opioid Morphine Equivalent	
OME Intraop (mg/kg)	7.2 ± 3.1
OME POD 0 (mg/kg)	0.4 ± 0.7
OME POD 1 (mg/kg)	1.0 ± 1.4
OME POD 2 (mg/kg)	1.2 ± 1.0
OME POD 3 (mg/kg)	1.0 ± 0.5
Total post-operative OME (mg/kg)	4.3 ± 3.2
Post-operative Epidural Infusion	2/14 (14%)
Post-operative Gabapentin	9/14 (64%)

Means with standard deviations are provided

Significant values are defined as *p* < 0.05

Mean case length was 418.9 min and estimated blood loss was 173 mL. No patient required a blood transfusion. Mean Foley catheter duration was 57 h and days to solid food was 1.1. Mean length of stay (LOS) was 4.1 days.

During the intraoperative and post-operative period, opioid morphine equivalent (OME) was measured and recorded. Mean OME use intraoperatively was 7.2 mg/kg and post-operatively, patients required an average of 4.3 mg/kg. Two (14%) patients had an epidural infusion post-operatively and nine (64%) patients used gabapentin on post-operative day 0.

### Revisions, complications, and clinical outcomes of ATVBT/PLST

Revisions, complications, and clinical outcomes for patients who underwent ATVBT/PLST are summarized in Table 4. Revision surgery occurred in 7/14 (50%) and cable breakage occurred in 6/14 (43%) of patients. Patient 1 is currently considering revision fusion after undergoing 2 revision procedures for cable breakage and lumbar overcorrection. Patient 2 underwent cable replacement and extension 2.1 years after initial surgery due to cable breakage. Patients 4 and 6 had revision surgeries due to lumbar overcorrection but have now been considered clinical successes with main thoracic and lumbar curves ≤ 30°. Patient 5 underwent a minor revision due to an unstable L4 set screw. Patients 7 (skeletally immature) and 9 (skeletally mature) underwent thoracic PSIF for thoracic curve progression, but the lumbar curves have remained stable with the original posterior tether. No patients treated with ATVBT/PLST have undergone or have indication for lumbar fusion.

**Table 4:** Complications and revision operations following anterior thoracic vertebral body tethering and posterior lumbar spine tethering

Patient number	Tether levels; subgroup type; apexes	Complication after surgery; time to complication (months); Levels broken; Location	Revision surgery; time since index surgery (months)	Clinical outcome
1	T4–T11 AT, T11–L3 PT; T7, L1	Cable breakage; 30; L2–L3; Below Lumbar Apex Lumbar overcorrection; 42; NA; Lumbar Curve	Cable replacement and L3–L4 extension due to L2–L3 cable breakage; 34 Release of T11–T12 tether due to overcorrection; 45	Has not achieved skeletal maturity, although has indication for thoracic fusion
2	T5–T11 AT, T11–L3 PT; T9, L2	Cable Breakage; 23; L2–L3; Below Lumbar Apex	Cable replacement and L3–L4 extension due to L2–L3 cable breakage; 25	Main thoracic curve > 30 degrees. Successful correction of lumbar curve with posterior tether
3	T5–T11 AT, T12–L4 PT; T9, L2	Cable breakage; 33; L3–L4; Below Lumbar Apex	None^a^	Clinical success without revision
4	T5–T12 AT, L1–L4 PT; T9, L3	Lumbar overcorrection; 2; NA; Lumbar Curve Lumbar overcorrection; 27; NA; Lumbar Curve	Revision of lumbar tether; 3 Removal of lumbar tether; 29	Clinical success following two revision surgeries
5	T4–T11 AT, T11–L3 PT; T6, L2	Unstable L4 set screw; 15; NA; NA Cable Breakage; 31; NA; Thoracic (T9–T10, T10–T11); Below Thoracic Apex	Replacement of L4 set screw; 15	Main thoracic and lumbar curve > 30 degrees
6	T4–T12 AT, T12–L3 PT; T8, L3	Lumbar overcorrection; 6; NA; Lumbar curve	Cable replaced (no breakage) but T12–L1 lumbar tether not applied; 6	Clinical success following one revision surgery
7	T5–T12 AT, T12–L4 PT; T8, L3	Progression of thoracic curve; 9; NA; Thoracic Curve	T3–T12 posterior spinal fusion; 12	Main thoracic curve correction with PSF, but has not achieved skeletal maturity
8	T5–T12 AT; L1–L4 PT; T9, L3	Possible cable breakage; 42; T12–L1; Above Lumbar Apex	None^a^	Clinical success without revision
9	T4–T11 AT; T11–L3 PT; T6, L2	Progression of thoracic curve; 40; NA; Thoracic curve	T1–L1 posterior spinal fusion; 40	Main thoracic curve correction with PSF. Successful lumbar correction with posterior tether
10	T6–T12 AT; T12–L4 PT; T10, L3	Possible cable breakage; 37; T10–T11; below Thoracic Apex	None^a^	Main thoracic curve > 30 degrees. Successful correction of lumbar curve with posterior tether
11	T6–T12 AT; T12–L4 PT; T10, L2	None	None	Clinical success without revision
12	T5–T12 AT; T12–L4 PT; T8, L2	None	None^a^	Clinical success without revision
13	T5–T11 AT; T12–L4 PT; T8, L2	None	None	Main thoracic curve > 30 degrees. Successful correction of lumbar curve with posterior tether
14	T5–T11 AT, T11–L4 PT; T8, L1	None	None	Clinical success without revision

Clinical success was defined as a patient who reached skeletal maturity with a ≤ 30° curve magnitude at most recent post-operative visit

^a^Patients 3, 8, 10, and 12 have suspected cable breakage but do not have indication for surgery given successful correction and skeletal maturity

### Clinical success

At most recent follow-up, 12 patients achieved skeletal maturity as defined by Risser stage 4/5 and closed triradiate cartilage. Among these patients, one underwent PSIF. Among the 11 patients who did not undergo PSIF, 10 patients (91%) achieved ≤ 30° correction of their lumbar curve with posterior tethering and 7 (64%) of these patients have also achieved ≤ 30° correction of their main thoracic curve with anterior tethering. Therefore, among the patients who did not require PSIF and were Risser 4 or 5 at most recent follow-up, 7/11 (64%) achieved clinical success with ≤ 30 correction of both their main thoracic and lumbar curves.

### Patient-reported outcomes

SRS-22r data are summarized in Table 5. SRS-22r was available at most recent follow-up for 13 of 14 patients. One patient did not participate because of a cancer diagnosis and treatment. Mean total score following ATVBT/PLST was 4.2 ± 0.4.

**Table 5:** SRS-22 at the time of most recent follow-up

SRS-22 Domain	ATVBT/PLST (*N* = 13)
Function	4.8 ± 0.3 (4.2–5)
Pain	4.0 ± 0.6 (2.6–4.4)
Self-Image	4.4 ± 0.5 (3.8–5)
Mental Health	3.5 ± 0.7 (2.8–4.8)
Satisfaction	4.3 ± 0.6 (3.5–5)
Total	4.2 ± 0.4 (3.5–4.8)

Means and standard deviations are provided, with range in parentheses

## Discussion

This is the first study to describe a combined anterior and posterior tethering approach for skeletally immature patients with AIS. We included 14 skeletally immature patients who underwent ATVBT/PLST and had a minimum 2-year follow-up.

Patients undergoing ATVBT/PLST achieved 51% main thoracic and 59% thoracolumbar/lumbar percent curve corrections at 1st post-operative visit and maintained this correction at the most recent post-operative visit with an average 43% main thoracic and 60% thoracolumbar/lumbar curve reduction. Mean curve correction is consistent with successful spine tethering reported by other studies [2, 4, 8, 9]. Additionally, ATVBT/PLST patients had a mean SRS-22 score of 4.2 which also is consistent with prior spine tethering and PSIF reports [2, 8].

As a principle, it is important to report all results, including if success rate is low or even in the event that results are negative. In our ATVBT/PLST group, 43% had cable breakage at an average of 2.7 years (2–3.5 years). Cable breakage was similar in both the main thoracic and lumbar curves. In the 23 cases reported by Newton et al., 44% had cable breakage, while Hoerneschmeyer et al. reported a rate of 54% at a minimum of 2 years after operation [8, 9]. A more recent study of single and double anterior lumbar tethers reported a cable breakage rate of approximately 73% [30]. It has become clear that the polyethylene terephthalate cable is the Achilles heel of spine tethering, given the rates of breakage [8]. Although 43% of patients in our cohort had cable breakage, it is important to recognize that some patients do not require revision surgery following cable breakage—as seen in four of our patients—because curve progression is not significant at the level(s) of breakage.

Newton et al*.* reported a revision rate of 30% in 23 patients, three of which were PSIF due to curve progression [8]. In the 29-case cohort reported by Hoerneschmeyer et al., rate of revision was lower at 21% with two patients progressing to PSIF [9]. The most common reasons for revision in our cohort were lumbar overcorrection, showing the concept validity and potency of the procedure in the lumbar spine. Previous literature by Miyanji et al*.* attributed lumbar overcorrection to patients with an open triradiate cartilage [7]. All of our patients who had lumbar overcorrection had an open triradiate cartilage [7].

An up to 50% revision rate means that half of patients will benefit from the procedure. For a novel procedure, there is value in recognizing the success rate at all time points in the procedure’s lifespan. This will aid surgeons in refining indications and techniques, which in turn will raise the success rate. Furthermore, if a procedure is low morbidity with potentially high reward, there can be a greater tolerance of failure. PLST is low morbidity as the approach is after Wiltse [28, 29], so blood loss and tissue dissection are minimal,as opposed to a retroperitoneal approach with or without a thoracotomy and diaphragmatic takedown for anterior tethering. Compared with fusion, there is no joint excision, no decortication, half the implants, less blood loss, and no graft agents. However, we realize we do not have data in this study to support these claims. Perhaps, the best statement to the lower morbidity is that we have performed isolated PLST when indicated as an outpatient procedure. Furthermore, there is significant value to conservation of spine motion, as opposed to its elimination [19–22].

Defining clinical success for our cohort of patients was based upon skeletal maturity, ≤ 30° residual magnitude of thoracic and lumbar curves, and avoidance of PSIF. In our cohort, we defined Risser ≥ 4 patients as skeletally mature. Progression after Risser 4 is critical in decision-making for braced curves. At our institution, we have not seen growth modulation, relevant to tethered curves, after Risser 4.

We have followed the early literature’s definition of success for spine tethering, namely, a mobile spine with up to 30° of deformity [31–33]. If a tether is a temporary intervention to conserve spine motion for a child during growth, while keeping the curve < 50°, then leaving the child with a curve of 30° is a success, as it would be for a brace. If a tether has to support the spine regardless of maturity, then barring cable breakage there will be no progression and no concern for worsening deformity into adulthood. With this in mind, 8/12 (67%) skeletally mature patients avoided fusion and were considered clinical successes with ≤ 30° magnitude of both main thoracic and lumbar curves at a minimum 2-year follow-up. One skeletally immature patient underwent thoracic fusion 12 months after the index operation. One skeletally mature patient underwent thoracic fusion 3.3 years following index operation. No patient has received or has indication for fusion of the lumbar spine.

Our mean EBL (mean 173 mL) is consistent with published literature for VBT [7, 8, 34, 35]. A functional assessment of blood loss is that none of our spine tethering patients has required a transfusion. Furthermore, no spine tethering patient has had an infection, screw failure or neural signal change, supporting that from a perioperative perspective the procedure is a safe alternative to fusion.

This study has limitations. Given the novelty of the procedure, our study is limited by sample size and retrospective design. While we have a minimum 2-year follow-up on all patients, we recognize that medium- and long-term follow-ups are essential to understanding a condition and a procedure. Such follow-up should be through maturity—which may be defined by Risser 5, by the distal physes of ulna and radius, or other radiographic method (*e.g.,* of Sanders)—when growth can deform the spine and thereby challenge any system that would resist such deformation. Long-term follow-up will give experience and perspective that can aid comparison with other methods of treatment. Long-term follow-up also will expose unanticipated consequences (such as the health of squeezed and partially immobilized intervertebral discs), bring focus to technical issues such as cable failure, and determine whether the intervention is temporary during growth (like a brace—in which case cable breakage may not matter and may even be an advantage) or must be durable as long as possible.

In conclusion, ATVBT/PLST is a potential alternative to spine fusion for select immature patients with AIS at a minimum 2-year follow-up. ATVBT/PLST potentially offers motion conservation at the cost of a higher revision rate. As we and others continue to study and report results, indications and techniques will be refined, which in turn will improve outcomes of this procedure.

## Data Availability

The data that support the findings of this study are not openly available due to reasons of sensitivity and are available from the corresponding author upon reasonable request.
